# Testing the psychometric characteristics of EQ-5D-5L and respiratory bolt-ons using a sample of the Australian population

**DOI:** 10.1007/s11136-024-03817-7

**Published:** 2024-10-26

**Authors:** Mina Bahrampour, Slavica Kochovska, David C. Currow, Rosalie Viney, Brendan Mulhern

**Affiliations:** 1 Centre for Health Economics Research and Evaluation (CHERE), University of Technology Sydney, Sydney, NSW Australia; 2 Faculty of Science, Medicine and Health, University of Wollongong, Wollongong, NSW 2522 Australia; 3 Faculty of Health, Improving Palliative, Aged and Chronic Care through Clinical Research and Translation (IMPACCT), University of Technology Sydney, Sydney, NSW Australia

**Keywords:** Respiratory, Bolt-on, Validity, Health related quality of life

## Abstract

**Purpose:**

The EQ-5D has been used to assess health related quality of life (HRQoL) in respiratory conditions. However, the core descriptive system may not be sensitive to all the HRQoL impacts of these conditions. To increase the sensitivity of the descriptive system, two respiratory specific bolt-ons, have been developed. Psychometric assessment of the bolt-ons in comparison to other validated instruments is required to facilitate their use. Therefore, the aim of this study is to test the psychometric characteristics of the EQ-5D-5L + R using a large dataset collected in Australia.

**Methods:**

A cross-sectional online survey was used to recruit adult respondents (≥ 18 years) representative of the Australian population. Descriptive and psychometric analyses were used to understand the performance of the EQ-5D-5L and bolt-ons in comparison to other validated instruments. The construct validity was estimated using correlations. Known-group validity was tested to determine the sensitivity of the instruments to differences across different severity groups.

**Results:**

Overall 10,033 respondents (52% female) completed the survey, of which 300 had a respiratory condition. There were moderate to high correlation between bolt-ons with EQ-5D-5L and WHODAS. The EQ-5D-5L + bolt-ons slightly reduced the ceiling effect in comparison to the EQ-5D-5L. The effect size was larger for people with respiratory conditions than people who did not have a respiratory condition.

**Conclusion:**

The results show that adding the respiratory bolt-on to the EQ-5D-5L might slightly improve the instruments descriptive sensitivity. The choice of bolt-on may be driven by whether overall problems or limitations are being measured.

**Supplementary Information:**

The online version contains supplementary material available at 10.1007/s11136-024-03817-7.

## Background

Respiratory problems, such as chronic obstructive pulmonary disease (COPD), are a leading cause of morbidity and mortality worldwide [1]. These conditions can significantly impact a person’s quality of life, leading to limitations in daily activities, reduced social interactions, and psychological distress. The EQ-5D is a generic instrument used to measure health-related quality of life (HRQoL) across different populations and healthcare settings. It includes five dimensions: mobility, self-care, usual activities, pain/discomfort, and anxiety/depression. The EQ-5D dimensions were initially described in three levels of severity (EQ-5D-3L). Subsequently, two extra levels were added to increase the sensitivity of the instrument and reduce the ceiling effect (EQ-5D-5L).

The EQ-5D instruments have been widely used in population studies and in the assessment of conditions that impact breathlessness [2–5]. Even though it was found that EQ-5D was a reliable instrument in some conditions such as COPD and asthma, its ability to distinguish between the milder stages of these conditions was limited. Also, a weak correlation between the EQ-5D and the clinical disease specific outcomes was indicated. Even though some impacts of persisting breathlessness may be picked up by EQ-5D dimensions such as usual activities and pain/discomfort [6–8], respiratory problems are often under-represented. The core descriptive system does not directly capture all the HRQoL impacts of respiratory conditions. This has led to questions about the sensitivity of EQ-5D in these conditions [9, 10].

Adding a bolt on was suggested as a solution to increase the sensitivity of the EQ-5D, for different health conditions [11–14]. Studies have identified gaps in the EQ-5D and the impact of adding bolt-ons for certain condition [15] such as hearing or cognition [16–18]. To improve the responsiveness of the EQ-5D in respiratory conditions, there have been bolt-ons developed for EQ-5D-5L that focus on assessing the impacts of breathlessness and respiratory conditions [19].

The respiratory bolt-ons are additional items that specifically addresses respiratory symptoms and limitations in activities related to breathing, such as shortness of breath, coughing, and wheezing. The performance of the breathlessness bolt-ons (EQ-5D-5L + Rs) have recently been tested in a COPD population [20]. Value sets for the respiratory bolt-ons based on the preferences of the Dutch population has been developed. It was found that by adding the respiratory bolt-on to the EQ-5D-5L, the performance of the utility instrument had a modest improvement in clinical sub-groups, and clinical indicators correlated slightly more with the EQ-5D-5L + R utilities [20].

Given the limited research in this area, further evidence is needed to evaluate the psychometric performance of the EQ-5D-5L and its respiratory bolt-ons in detecting breathlessness, especially compared to other validated instruments and clinical indicators. The aim of this study was to assess the psychometric properties of the EQ-5D-5L for respiratory conditions, specifically investigating whether adding respiratory bolt-ons enhances its sensitivity and responsiveness. The study focused on determining if these bolt-ons improve the instrument’s ability to detect breathlessness, compared to other established measurement tools.

## Methods

The study was conducted using a dataset collected online in Australia exploring the prevalence of breathlessness in the population. The dataset included the EQ-5D-5L and respiratory bolt-ons alongside World Health Organisation Disability Assessment Schedule (WHODAS) 2.0 and modified Medical Research Council (mMRC) breathlessness scale. Inclusion criteria for the current study were complete responses relating to these measures.

### Data

A cross-sectional online survey using the Qualtrics platform recruited 10,033 adult respondents (aged *≥* 18 years) representative of the Australian population by age, sex, state/territory of residence and rurality using the Australian 2016 Census [21]. Potential participants were invited by Qualtrics through its database of consenting, registered panel members which are selected from multiple sources to create a blended sample. Qualtrics has over 800,000 registered, consenting members. Recruitment quotas were set up for the survey’s four key demographic parameters, and potential respondents were screened out once each quota was filled with the required number. The plan for data gathering was to include the first 10,000 responses from individuals whose characteristics aligned with the demographics of each defined group, including age brackets, sex, state or territory of residence, and rurality. This was a community survey, with recruitment being independent of health or social service contact or utilisation.

The survey was piloted with members of the University of Technology’s Improving Palliative, Aged and Chronic Care through Clinical Research and Translation (IMPACCT) Consumer Advisory Group and minor wording and formatting changes were made to improve the survey’s comprehension and readability. The survey was then piloted with 110 participants to establish face validity before its full launch; no changes were made to the survey’s content and design as a result of the soft launch. Consent was obtained from participants at the time of joining the respondent panel and, again, at the time of participating in the survey.

The ethics approval was obtained from the Human Research Ethics Committees of the University of Technology Sydney (UTS HREC ETH20-5114).

### Instruments included in the study

EQ-5D-5L and Respiratory bolt-ons (EQ-5D-5L + Rs) and Visual Analogue Scale (VAS):

The Australian version of the EQ-5D-5L, and the associated EQ-VAS was used. The structure of the survey was the EQ-5D-5L instrument followed by bolt-on dimensions and then the EQ-VAS. The wording of the questions was as set out in the development paper [19]: R1 as Limitations in physical activities due to shortness of breath (e.g., climbing stairs, going for a walk, carrying things, gardening) and R2 as Breathing problems (e.g., shortness of breath, wheezing, coughing, sputum) with the same severity levels as the EQ-5D-5L. The Dutch utility values were generated using the opinion of general population and it was from − 0.65 to one for R1 and between − 0.78 and one for R2.

#### The modified medical research council (mMRC) breathlessness scale

The mMRC breathlessness scale [22] is a self-rated, ordinal measure that estimates the degree of exertion before breathlessness limits function. It is measured on a scale 0–4, with: 0 = no breathlessness except on strenuous exercise; 1 = shortness of breath when hurrying on the level or walking up a slight hill; 2 = walks slower than people of same age on the level because of breathlessness or has to stop to catch breath when walking at their own pace on the level; 3 = stops for breath after walking ∼100 m or after few minutes on the level; and 4 = too breathless to leave the house, or breathless when dressing or undressing [22].

Self-reported primary cause of breathlessness was also sought using a multiple-choice question. Respondents who chose 1–4 on the mMRC scale were asked to identify the condition that was causing their breathlessness (one response only): (1) lungs, (2) heart, (3) disorder of nerves or muscles, (4) cancer, (5) other [please specify] (6) don’t know, and (7) prefer not to say. As this was a self-reported index, it couldn’t be used as a clinical condition detection.

#### The world health organisation disability assessment schedule (WHODAS) 2.0

The WHODAS is a generic assessment instrument for health and disability [23]. This instrument is used across all conditions including mental, neurological, and addictive disorders. It can be used in both general and clinical population settings. The instrument has 12 items that cover six dimensions: cognition, self-care, mobility, getting along, life activities, and participation. Each item has 5 levels: “none”, “mild”, “moderate”, “severe”, and “extreme” [24]. To score the WHODAS, responses are assigned numbers from 0 to 4, with ‘none’ corresponding to zero and ‘extreme’ to four. The WHODAS score is calculated by summing all item responses [23]. In the current sample, Cronbach’s alpha for the total score was 0.94 (and for each domain: cognition = 0.78, mobility = 0.75, self-care = 0.76, getting along = 0.7, life activities = 0.7, and participation = 0.76).

### Data analysis

Descriptive statistics (number of participants, number of missing data, mean, standard deviation, median, and range) for the EQ-5D-5L index and VAS scores, and WHODAS were assessed. The mMRC scale was used to assess the severity of the respondent’s breathlessness. There were no missing data regarding the EQ-5D-5L index and VAS scores, respiratory bolt-ons, WHODAS nor mMRC.

#### Ceiling and floor effect

Floor and ceiling effects were calculated by identifying the proportion of participants that either had the lowest or the highest possible scores for the EQ-5D-5L and WHODAS. Ceiling and floor effects were compared between EQ-5D-5L and EQ-5D-5L + Rs to test the impact of adding bolt-on to descriptive systems. Absolute changes in ceiling effect is measured by the difference between the proportion of “no problem” responses and relative changes is measured by Eq. 1 [25].

1 $$\frac{{ceiling5L - ceilingR}}{{ceiling5L}} \times 100$$

#### Convergent validity

Convergent validity shows if the instruments or variables have the same construct. Spearman correlation was examined to determine the relationship between the instruments. Correlations between the EQ-5D-5L + R dimensions were examined. Coefficients from 0.1 to 0.29 were considered to be weak, 0.3 to 0.49 as moderate, and correlations of 0.5 or above as strong [26].

#### Known-groups analyses

Known-groups validity is the ability of an instrument to distinguish among respondents based on their disease severity. Known-groups validity was assessed for the EQ-5D-5L index and VAS scores, and WHODAS scores by grouping respondents based on their mMRC and smoking history. Smoking (former or current smoker versus no smoking history) was selected as a known group indicator as a lot of respiratory diseases such as COPD and lung conditions are caused and worsen by smoking [27]. Effect size was used to explore the size of the observed difference of summary scores between the different groups. The effect size is the mean difference between adjacent severity classes divided by the standard deviation of the milder class. The effect size emphasises the difference between the groups without confounding with the sample size. A larger effect size signifies better discriminating ability. Using Cohen’s d, a rule of thumb for effect size is that less than 0.2 is considered a small effect, 0.5 is a medium effect and 0.8 or higher is large [28].

## Results

### Demographics

Overall, 10,033 respondents (∼ 52% female) completed the survey and all were included in the analyses having provided complete data on EQ-5D-5L index and VAS scores, respiratory bolt-ons, WHODAS and mMRC. Mean age for females was 40 years and for males was 51.6 years. About 25% of the sample population were aged between 18 and 29 years. Among the people who had an mMRC of one and above, ∼ 36% reported lung problems as the primary cause of breathlessness. Respondent’s characteristics can be viewed in Table 1.

**Table 1:** Sample characteristics (*n* = 10,033)

Gender	*n*	%
Male	4,757	47.41
Female	5,212	51.95
Other	64	0.64
**Age category**		
18–29	2,522	25.17
30–39	1,891	18.87
40–49	1,449	14.46
50–59	1,316	13.13
60–69	1,536	15.33
70–79	1,103	11.01
80 or more	204	2.04
Missing	12	0.12
**Region**		
Metro	5,983	59.63
Regional	4,050	40.37
**Smoking Status**		
Current smoker	2,229	22.2
Former smoker	2,775	27.7
Never smoked	4,906	48.9
Prefer not to say	123	1.2
**Condition (for mMRC > = 1)**	*N* = 4245	
Lungs condition (e.g. emphysema, smoking, asbestosis, work related, asthma)	1,530	36.04
Heart condition (e.g. ischaemic heart disease, valve disease, abnormal heart rhythms)	515	12.13
Disorder of nerves or muscles condition (e.g. motor neurone disease, multiple sclerosis)	178	4.19
Cancer	52	1.22
Other	739	17.41
Don’t know	1,139	26.83
Prefer not to say	92	2.17

### Descriptive analysis

In total, 300 of the participants had an mMRC score of 3 or 4 indicating minimal exertion generated limiting breathlessness. More than 50% of the participants reported no problems (mMRC = 0). Mean VAS for this sample was 73. “No problem” was the most chosen severity level for the EQ-5D-5L (and + Rs), with “pain/discomfort” having the lowest proportion of responses in this category and “self-care” having the highest. Among the bolt-ons R1 “limitations in physical activities” had higher percentage of “no problem” compared to the R2 “breathing problem” dimension.

**Fig. 1:**
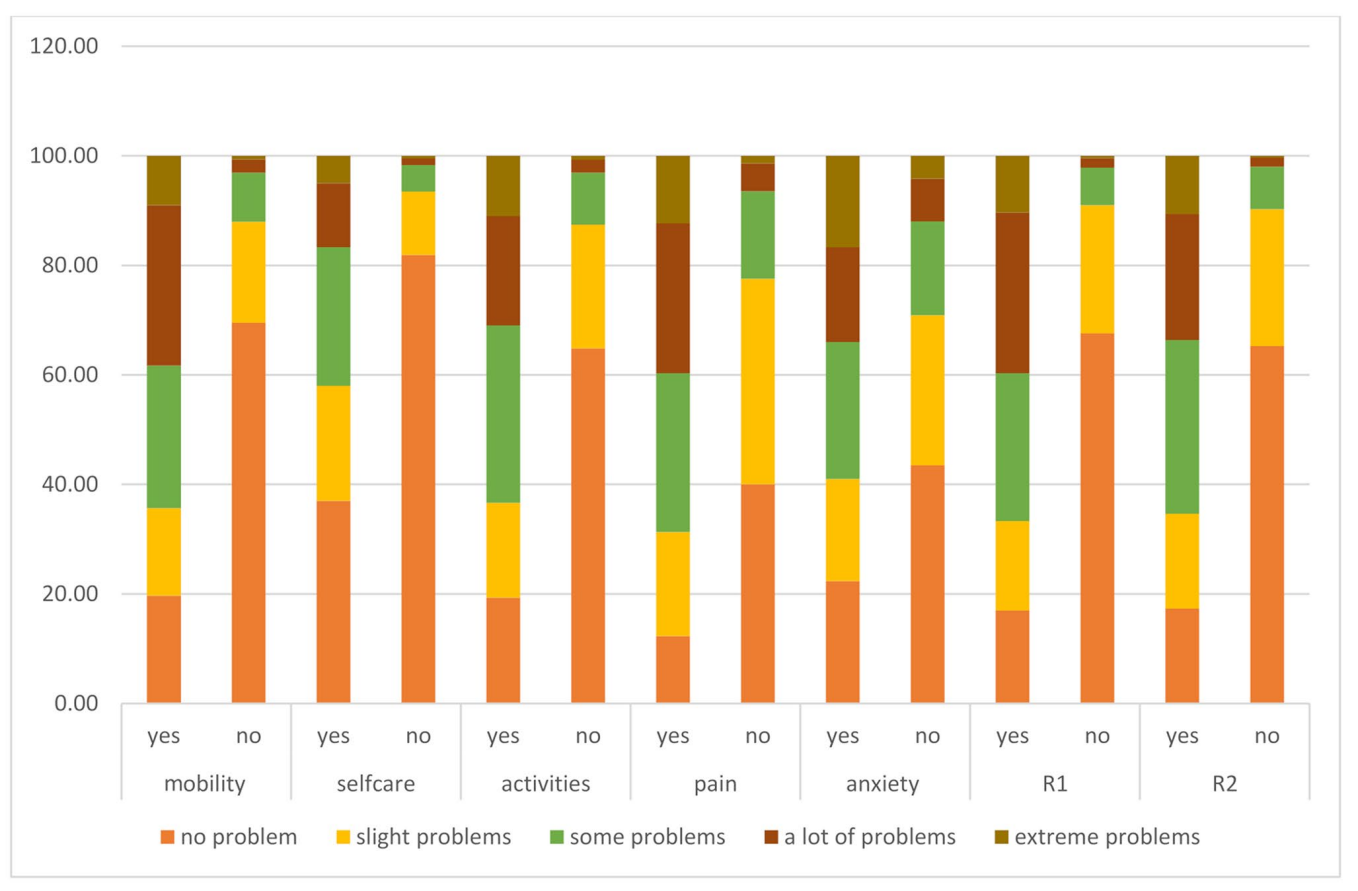
Percentage of respondents per level for each EQ-5D-5L dimension

Figure 1 shows an overview of the percentage of respondents per level for each EQ-5D-5L dimension in subgroups that present people who have versus do not have a respiratory condition. Having “no problems” always has the higher percentage in the no condition group. Chi square *P* values (< 0.001) were significant for all the domains.

### Ceiling and floor effect

In total, 843 health profiles out of 3125 (27%) were reported for the EQ-5D-5L. For EQ-5D-5L + R1 and EQ-5D-5L + R2, 1511 (9.7%) and 1492 (9.5%) health profiles were reported respectively. The EQ-5D-5L + R1 and EQ-5D-5L + R2 had a very marginal reduced ceiling effect in comparison to the EQ-5D-5L. EQ-5D-5L + R2 had the lowest ceiling effect among the instruments. Relative change from EQ-5D-5L for “limitations in physical activities” was 3.83% and 6.15% for “breathing problem”. Absolute change in ceiling effect for Bolt-on R1 was 0.89% and it was 1.43% for R2. There was no difference between the ceiling effect of EQ-5D-5L and EQ-5D-5L + R1 in the sample with respiratory condition this suggests that the EQ-5D-5L performs similarly to the EQ-5D-5L + R1 at picking up the issues in respiratory group (Table 2).

**Table 2:** Ceiling and floor effects

		No problem | None	Absolute change	Relative change	Worst health state
All (*N* = 10,033)	EQ-5D-5L	23.24%			0.14%
	EQ-5D-5L + R1	22.35%	0.89%	3.83%	0.13%
	EQ-5D-5L + R2	21.82%	1.43%	6.13%	0.19%
	WHODAS	23.48%			0.08%
With Respiratory condition (*N* = 300)	EQ-5D-5L	6.67%			2.67%
	EQ-5D-5L + R1	6.67%	0	No change	2.33%
	EQ-5D-5L + R2	6.33%	0.34%	5%	2.33%
No Respiratory condition (*N* = 9,733)	EQ-5D-5L	23.75%			0.12%
	EQ-5D-5L + R1	22.83%	0.92%	3.89%	0.07%
	EQ-5D-5L + R2	22.30%	1.45%	6.10%	0.12%

### Convergent validity

The “Limitations in physical activities due to shortness of breath” bolt-on had a high correlation with “usual activities” from EQ-5D-5L (0.52) (Table 3) and “walking a long distance” from WHODAS (0.50) (Table 4). Other dimensions had moderate correlations. The “breathing problems” bolt-on had moderate correlation with almost all EQ-5D-5L and WHODAS dimensions (< 0.5). There was a strong correlation between the bolt-on dimensions (0.71).

**Table 3:** Correlation between EQ-5D and bolt-on dimensions

EQ-5D-5L + Rs	Mobility	Self-care	Usual activities	Pain| Discomfort	Anxiety |depression	Physical activities limitations	Breathing problems
Mobility	1.00						
Self-care	**0.59**	1.00					
Usual activities	**0.66**	**0.61**	1.00				
Pain| Discomfort	**0.59**	*0.46*	**0.60**	1.00			
Anxiety |depression	0.27	*0.33*	*0.44*	*0.41*	1.00		
Physical activities limitations	*0.48*	*0.44*	**0.52**	*0.45*	*0.39*	1.00	
Breathing problems	*0.47*	*0.44*	*0.48*	*0.45*	*0.39*	**0.71**	1.00

*The bold items indicate a strong correlation

**Table 4:** Correlation between EQ-5D and bolt-on and WHODAS items

WHODAS^i^\EQ-5D-5L + Rs	Mobility	Self-care	Usual Activities	Pain |discomfort	Anxiety |depression	Physical activities Limitations	Breathing problems
WHODAS 1	**0.55**	0.43	**0.51**	0.47	0.28	0.43	0.39
WHODAS 2	0.47	0.48	**0.56**	0.41	0.37	0.43	0.41
WHODAS 3	0.31	0.39	0.37	0.25	0.32	0.34	0.32
WHODAS 4	0.38	0.40	0.45	0.32	0.35	0.37	0.35
WHODAS 5	0.42	0.40	**0.54**	0.46	**0.55**	0.44	0.41
WHODAS 6	0.31	0.39	0.42	0.29	0.45	0.36	0.36
WHODAS 7	**0.59**	0.45	**0.54**	0.47	0.30	**0.50**	0.45
WHODAS 8	0.44	**0.58**	0.43	0.32	0.26	0.38	0.38
WHODAS 9	0.45	**0.60**	0.44	0.32	0.26	0.37	0.36
WHODAS 10	0.29	0.36	0.39	0.30	0.49	0.36	0.34
WHODAS 11	0.29	0.37	0.38	0.29	0.46	0.35	0.34
WHODAS 12	0.43	0.47	**0.54**	0.40	0.41	0.42	0.40

*The bold items indicate a strong correlation

^i^WHODAS (1) Standing for long periods such as 30 min? WHODAS (2) Taking care of your household responsibilities? WHODAS (3) Learning a new task, for example, learning how to get to a new place WHODAS (4) How much of a problem did you have joining in community activities (for example, festivities, religious or other activities) in the same way as anyone else can WHODAS (5) How much have you been emotionally affected by your health problems? WHODAS (6) Concentrating on doing something for ten minutes? WHODAS (7) Walking a long distance such as a kilometre [or equivalent]? WHODAS (8) Washing your whole body? WHODAS (9) Getting dressed? WHODAS (10) Dealing with people you do not know? WHODAS (11). Maintaining a friendship? WHODAS (12). Your day−to−day work

More information about instrument and bolt-on correlations can be found in Appendix Tables 1 and 2.

### Known group validity

#### Utility values using Dutch algorithm

Utility decrements for the respiratory dimension are highest for the more severe levels (mMRC 3 or 4). Mean utility value for the bolt-on instruments is almost the same when mMRC is equal to zero but is lower for the “breathing problem” (Table 5). The EQ-5D-5L + Rs presented more differences in utility values between reparatory subgroups than the EQ- 5D-5L. Correlation between mMRC score and utility values for the EQ-5D-5L was 0.475, and it was 0.486 and 0.503 for the EQ-5D-5L + R1 and EQ-5D-5L + R2 respectively^1^.

**Table 5:** Mean utility value for each mMRC level

mMRC level (*n*)	EQ-5D-5L mean (CI)	EQ-5D-5L + R1 mean (CI)	EQ-5D-5L + R2 mean (CI)	VAS - Mean (CI)
0 (5788)	0.83 (0.83,0.84)	0.85(0.84,0.85)	0.88 (0.84,0.85)	79.39 (78.91,79.86)
1 (3139)	0.70 (0.69,0.71)	0.71 (0.70,0.72)	0.71 (0.70,0.72)	67.81(67.08,68.54)
2 (806)	0.48(0.46,0.50)	0.48 (0.46,0.50)	0.47 (0.45,0.49)	56.37 (54.77,57.95)
3 (239)	0.35(0.31,0.40)	0.33 (0.28,0.38)	0.29 (0.24,0.34)	47.55 (44.38,50.71)
4 (61)	0.34(0.21,0.48)	0.29 (0.15,0.44)	0.26 (0.10,0.42)	52.25(44.42, 60.07)
no respiratory condition (9733)	0.76 (0.76,0.77)	0.77 (0.77, 0.78)	0.77 (0.77,0.78)	73.74(73.33, 74.17)
with respiratory condition (300)	0.35(0.31 0.40)	0.32 (0.28 0.37)	0.28 (0.23 0.33)	48.50(45.52,51.49)

The effect size was used to compare the discriminatory power of the instruments (Table 6). Results indicate a large effect size when comparing the utility values of people with respiratory condition (if mMRC is equal to 3 or 4) and people who did not have a respiratory condition (if mMRC is equal to 0 or 1 or 2). The largest effect size was related to the “breathing problem” dimension. The effect size was also measured for respondent with a history of smoking (either former or current smokers) and non-smokers, there was a very slight difference between the variables. (Appendix 4 shows the effect size by mMRC levels.)

**Table 6:** Known group validity effect size and mean values

		Having respiratory condition		Smoker (*n* = 123 missing)	
		Yes (*N* = 300)	No (*N* = 9733)	Yes (*N* = 5004)	No (*N* = 4906)
***EQ-5D-5L***	Effect size	1.64		0.31	
	Mean utility score (SD)	0.35 (0.39)	0.76 (0.24)	0.71 (0.28)	0.78 (0.23)
**EQ-5D-5L + R1**	Effect size	1.75		0.31	
	Mean utility score (SD)	0.32 (0.43)	0.77 (0.25)	0.72 (0.29)	0.80 (0.23)
**EQ-5D-5L + R2**	Effect size	1.93		0.32	
	Mean utility score (SD)	0.28 (0.46)	0.77(0.24)	0.72 (0.29)	0.80 (0.23)
***VAS***	Effect size	1.19		0.30	
	Mean utility score (SD)	48.50 (48.50)	73.75(21.08)	69.84 (23.25)	76.30 (19.33)
*WHODAS	Effect size	1.42		0.32	
	Mean utility score (SD)	33.66 (10.84)	20.50 (9.18)	22.32 (10.14)	19.35 (8.52)

*For WHODAS sum score was used

## Discussion and conclusion

This study explored the validity of the EQ-5D-5L and the recently developed respiratory bolt-on dimensions that assess “limitations in physical activities due to shortness of breath (R1)” (also mentioned as “physical activity limitation”), and “breathing problems (R2)”. The evidence showed that adding a respiratory dimension does not make a significant impact on the EQ-5D, yet it does result in a slight improvement in performance of the instrument specially for people with respiratory conditions. By adding bolt-ons the ceiling effect decreased very slightly for the R2 bolt-on compared to the core descriptive system. This implies that adding the R2 bolt-ons has a marginal improvement in the descriptive sensitivity of the instrument especially for people with respiratory conditions.

The instruments showed a moderate correlation. Among the two respiratory bolt-on dimensions, “physical activity limitation” had strong correlation with some dimensions from EQ-5D-5L and WHODAS, while the “breathing problem” had a moderate correlation with all dimensions from EQ-5D-5L and WHODAS. The bolt-on R2 did not have high correlation with other dimensions of other instruments which shows that there is a less direct relationship between this dimension and other dimensions compared to the R1 bolt-on. The bolt-on developers indicated that when estimating utility values, R2 dimension showed more inconsistencies on other dimensions compared with R1, inconsistencies were mostly related to levels 2 and 3 being same as level one This study suggests that R2 is the most appropriate respiratory bolt-on which is consistent with previous study [19].

Measuring the psychometric properties of these bolt-ons can help determine whether adding these dimensions enhances the measure’s validity and reliability. A bolt-on, when used at the measurement level, introduces a new item that captures constructs not included in the core EQ-5D instrument. In a valuation study, a value set incorporating these new dimensions can provide utility values that reflect trade-offs across both the core and new dimensions. Consequently, if such value sets are available, the inclusion of bolt ons may result in more accurate utility values for health economic evaluations and lead to better decision-making in clinical trials and health technology assessments.

Adding bolt-ons changed the utility values, especially for people who were experiencing respiratory problem (mMRC breathlessness scales 3 or 4); EQ-5D-5L + R utility values were lower compared with the core instrument without bolt-on dimension, this was also seen in the cognitive bolt-on study [29]. Similar to previous bolt-on studies, our research also showed that adding a bolt-on dimension does not make change in the better levels of health and the utility reduction is more seen in worst health states [13, 20, 30, 31].

Correlation between mMRC score and utility has been about 0.5 which is consistent with previous studies [20, 32]. Correlation between mMRC score was higher for the EQ-5D-5L + R2. These demonstrate that adding bolt-ons leads to larger utility decrements. The utility decrements for the respiratory dimension are highest for the more severe levels, which is consistent with the standard EQ-5D-5L dimensions.

Although the bolt-on instruments are not suggested to be used as a stand-alone instrument at this stage, they can be used alongside the EQ-5D-5L to estimate a so called ‘‘bolt-on’’ Quality Adjusted Life Years (QALYs) [18, 19]; in addition to the calculation of the standard QALYs to show the potential change in treatment impact when a condition specific dimension or instrument is included in the economic evaluation. The choice of bolt-on may be driven by whether overall problems or limitations are being measured. It is worth to note that by adding a new dimension (bolt-on) to EQ-5D and developing new value set, will reduce the EQ-5D outcomes comparability among different conditions.

The current dataset does not allow for an assessment of the EQ-5D-5L + R instrument’s responsiveness as it was only measured once. Further research is required to investigate whether the EQ-5D-5L + Rs will be more responsive to changes in patients’ health status longitudinally. Using existing data is an informative way to understand the measurement performance of bolt-ons in comparison to the core descriptive system and other HRQoL instruments.

Adding bolt-ons did not significantly improve the measure for respiratory conditions. The psychometric analysis, which is sample-specific, did not show a significant difference between the use of bolt-ons and the core EQ-5D instrument alone. This may be attributed to the relatively low proportion of participants with cardiorespiratory conditions in this general population survey. Future studies could test the bolt-ons and the psychometric performance in a group with more participants who have breathing problems. A limitation of this study was the lack of clinical measurements available to assess breathing function (e.g. FEV1) and clinical variables which would be helpful to compare different clinical condition groups. Another limitation is that the Dutch valuation algorithm was used for the Australian dataset as there were no Australian utility values available for the respiratory bolt-ons.

Overall adding respiratory bolt-ons to the EQ-5D resulted in a slight improvement in the instruments ability to detect subtle changes making it slightly more sensitive for people with respiratory conditions. Adding a respiratory bolt-on could potentially improve the responsiveness of the EQ-5D for assessing people with breathlessness.

## Electronic supplementary material

Below is the link to the electronic supplementary material.

Supplementary Material 1

Supplementary Material 2

## Footnotes

1 Mean and effect size were checked by a subsample as a sensitivity analysis (appendix 3).
