# Supplementary material for: Testing the psychometric characteristics of EQ-5D-5L and respiratory bolt-ons using a sample of the Australian population
Source: Qual Life Res. 2024 Oct 26;34(2):395–403. doi: 10.1007/s11136-024-03817-7 (PMC11865160; doi:10.1007/s11136-024-03817-7)
Supplement: Supplementary file 2 — Supplementary Material 2 [file 11136_2024_3817_MOESM2_ESM.docx]

**Appendix:**

***Appendix 1: Spearman correlation between EQ-5D+R and EQ-VAS and WHODAS***

|  |  | variables | Spearman correlation (CI 95%) |
| --- | --- | --- | --- |
| EQ-VAS | All | EQ-VAS and EQ-5D-5L | -0.59 ( -0.60, -0.57) |
|  |  | EQ-VAS and EQ-5D-5L+R1 | -0.60 ( -0.61, -0.58) |
|  |  | EQ-VAS and EQ-5D-5L+R2 | -0.59 ( -0.61, -0.58) |
|  | With Respiratory condition | EQ-VAS and EQ-5D-5L | -0.47 (-0.57, -0.36) |
|  |  | EQ-VAS and EQ-5D-5L+R1 | -0.48 (-0.58, -0.38) |
|  |  | EQ-VAS and EQ-5D-5L+R2 | -0.49 (-0.59, -0.39) |
|  | No Respiratory condition | EQ-VAS and EQ-5D-5L | -0.57 (-0.59, -0.56) |
|  |  | EQ-VAS and EQ-5D-5L+R1 | -0.58 (-0.60, -0.57) |
|  |  | EQ-VAS and EQ-5D-5L+R2 | -0.58(-0.60, -0.57) |
| WHODAS | All | WHODAS and EQ-5D-5L | 0.69 (0.68, 0.70) |
|  |  | WHODAS and EQ-5D-5L+R1 | 0.70 (0.69, 0.71) |
|  |  | WHODAS and EQ-5D-5L+R2 | 0.70 (0.69, 0.71) |
|  | With Respiratory condition | WHODAS and EQ-5D-5L | 0.58 (0.50, 0.65) |
|  |  | WHODAS and EQ-5D-5L+R1 | 0.58 (0.50, 0.65) |
|  |  | WHODAS and EQ-5D-5L+R2 | 0.57 (0.49, 0.64) |
|  | No Respiratory condition | WHODAS and EQ-5D-5L | 0.67 (0.66, 0.68) |
|  |  | WHODAS and EQ-5D-5L+R1 | 0.69 (0.68, 0.70) |
|  |  | WHODAS and EQ-5D-5L+R2 | 0.68 (0.67, 0.69) |

**Appendix 2: Cross tabulation of EQ-5D dimensions and bolt-on dimensions:**

|  |  |  | **Physical Activities Limitations** | | | | | **Breathing problems** | | | | |
| --- | --- | --- | --- | --- | --- | --- | --- | --- | --- | --- | --- | --- |
|  |  |  | no problems | slight problems | moderate problems | severe problems | unable | no problems | slight problems | moderate problems | severe problems | unable |
| Mobility | With  Respiratory condition | no problems | 37 | 9 | 9 | 3 | 1 | 34 | 13 | 8 | 3 | 1 |
|  |  | slight problems | 6 | 21 | 13 | 8 | 0 | 6 | 19 | 18 | 5 | 0 |
|  |  | moderate problems | 1 | 14 | 34 | 26 | 3 | 3 | 10 | 35 | 25 | 5 |
|  |  | severe problems | 5 | 3 | 21 | 43 | 16 | 5 | 8 | 29 | 31 | 15 |
|  |  | unable | 2 | 2 | 4 | 8 | 11 | 4 | 2 | 5 | 5 | 11 |
|  | No Respiratory condition | no problems | 5443 | 1139 | 161 | 15 | 8 | 5,266 | 1,274 | 203 | 20 | 3 |
|  |  | slight problems | 740 | 774 | 223 | 51 | 7 | 740 | 791 | 216 | 44 | 4 |
|  |  | moderate problems | 278 | 300 | 221 | 67 | 9 | 249 | 297 | 262 | 61 | 6 |
|  |  | severe problems | 90 | 58 | 45 | 32 | 7 | 75 | 57 | 65 | 28 | 7 |
|  |  | unable | 28 | 7 | 12 | 8 | 10 | 25 | 9 | 13 | 6 | 12 |
| Self-care | With  Respiratory condition | no problems | 44 | 23 | 28 | 11 | 5 | 37 | 27 | 28 | 17 | 2 |
|  |  | slight problems | 2 | 15 | 20 | 24 | 2 | 2 | 15 | 29 | 15 | 2 |
|  |  | moderate problems | 5 | 9 | 21 | 33 | 8 | 5 | 5 | 32 | 25 | 9 |
|  |  | severe problems | 0 | 1 | 12 | 17 | 5 | 4 | 5 | 6 | 11 | 9 |
|  |  | unable | 0 | 1 | 0 | 3 | 11 | 4 | 0 | 0 | 1 | 10 |
|  | No Respiratory condition | no problems | 6052 | 1566 | 309 | 36 | 11 | 37 | 27 | 28 | 17 | 2 |
|  |  | slight problems | 384 | 485 | 190 | 56 | 9 | 2 | 15 | 29 | 15 | 2 |
|  |  | moderate problems | 103 | 181 | 129 | 51 | 4 | 5 | 5 | 32 | 25 | 9 |
|  |  | severe problems | 27 | 36 | 28 | 25 | 7 | 4 | 5 | 6 | 11 | 9 |
|  |  | unable | 13 | 10 | 6 | 5 | 10 | 4 | 0 | 0 | 1 | 10 |
| Usual activities | With  Respiratory condition | no problems | 38 | 10 | 5 | 5 | 0 | 32 | 12 | 8 | 5 | 1 |
|  |  | slight problems | 4 | 19 | 18 | 11 | 0 | 5 | 15 | 23 | 8 | 1 |
|  |  | moderate problems | 5 | 13 | 39 | 36 | 4 | 6 | 18 | 39 | 29 | 5 |
|  |  | severe problems | 2 | 3 | 14 | 31 | 10 | 3 | 3 | 20 | 21 | 13 |
|  |  | unable | 2 | 4 | 5 | 5 | 17 | 6 | 4 | 5 | 6 | 12 |
|  | No Respiratory condition | no problems | 5270 | 916 | 104 | 13 | 10 | 5050 | 1093 | 152 | 14 | 4 |
|  |  | slight problems | 925 | 959 | 257 | 51 | 5 | 941 | 925 | 283 | 45 | 3 |
|  |  | moderate problems | 291 | 329 | 230 | 64 | 11 | 284 | 327 | 246 | 63 | 5 |
|  |  | severe problems | 71 | 56 | 59 | 36 | 5 | 62 | 67 | 61 | 32 | 5 |
|  |  | unable | 22 | 18 | 12 | 9 | 10 | 18 | 16 | 17 | 5 | 15 |
| Pain \| discomfort | With  Respiratory condition | no problems | 27 | 2 | 1 | 5 | 2 | 26 | 2 | 4 | 4 | 1 |
|  |  | slight problems | 12 | 19 | 14 | 12 | 0 | 10 | 20 | 19 | 8 | 0 |
|  |  | moderate problems | 8 | 18 | 37 | 21 | 3 | 8 | 19 | 38 | 17 | 5 |
|  |  | severe problems | 4 | 9 | 22 | 37 | 10 | 5 | 8 | 24 | 32 | 13 |
|  |  | unable | 0 | 1 | 7 | 13 | 16 | 3 | 3 | 10 | 8 | 13 |
|  | No Respiratory condition | no problems | 3451 | 369 | 59 | 14 | 3 | 3375 | 445 | 62 | 12 | 2 |
|  |  | slight problems | 2238 | 1177 | 203 | 29 | 8 | 2180 | 1199 | 239 | 35 | 2 |
|  |  | moderate problems | 671 | 552 | 267 | 55 | 8 | 604 | 606 | 297 | 39 | 7 |
|  |  | severe problems | 171 | 145 | 110 | 58 | 9 | 153 | 140 | 138 | 56 | 6 |
|  |  | unable | 48 | 35 | 23 | 17 | 13 | 43 | 38 | 23 | 17 | 15 |
| Anxiety \| depression | With  Respiratory condition | no problems | 29 | 8 | 14 | 10 | 6 | 29 | 12 | 11 | 12 | 3 |
|  |  | slight problems | 10 | 14 | 16 | 14 | 2 | 13 | 14 | 21 | 7 | 1 |
|  |  | moderate problems | 5 | 12 | 23 | 31 | 4 | 4 | 14 | 31 | 20 | 6 |
|  |  | severe problems | 5 | 8 | 17 | 19 | 3 | 5 | 4 | 19 | 20 | 4 |
|  |  | unable | 2 | 7 | 11 | 14 | 16 | 1 | 8 | 13 | 10 | 18 |
|  | No Respiratory condition | no problems | 3603 | 502 | 97 | 22 | 10 | 3515 | 599 | 100 | 20 | 0 |
|  |  | slight problems | 1704 | 785 | 140 | 31 | 6 | 1633 | 833 | 178 | 17 | 5 |
|  |  | moderate problems | 809 | 581 | 231 | 39 | 10 | 780 | 587 | 247 | 50 | 6 |
|  |  | severe problems | 293 | 274 | 135 | 52 | 2 | 271 | 269 | 161 | 49 | 6 |
|  |  | unable | 170 | 136 | 59 | 29 | 13 | 156 | 140 | 73 | 23 | 15 |

Appendix 3: sensitivity analysis for sample size N=600 by random resampling (no respiratory n=300 with respiratory n=300)

The results show a slight difference in the mean utility values and effect size.

| Respiratory | EQ-5D-5L mean (CI) | EQ-5D-5L+R1 mean (CI) | EQ-5D-5L+R2 mean (CI) | VAS - Mean (CI) |
| --- | --- | --- | --- | --- |
| No | 0.71 (0.71, 0.77) | 0.75 (0.72, 0.78) | 0.74 (0.71, 0.78) | 72.85 (70.44, 75.26) |

| Effect size (N=600) | ***Having respiratory condition*** | | ***Smoker (missing n=9)*** | |
| --- | --- | --- | --- | --- |
|  | Yes  N=300 | No  N=300 | Yes  N=348 | No  N=243 |
| ***EQ-5D-5L*** | 1.13 | | 0.37 | |
| **EQ-5D-5L+R1** | 1.18 | | 0.39 | |
| **EQ-5D-5L+R2** | 1.22 | | 0.42 | |
| ***VAS*** | 1.02 | | 0.42 | |
| ***WHODAS** | 1.22 | | 0.44 | |

Appendix 4: effect size moving mMRC levels.

| Effect size | ***mMrc level*** | | | |
| --- | --- | --- | --- | --- |
|  | ***1-2*** | ***2-3*** | ***3-4*** | ***3 – (4&5 combined) ^i^*** |
| ***EQ-5D-5L*** | 0.64 | 0.89 | 0.42 | -0.41 |
| **EQ-5D-5L+R1** | 0.63 | 0.93 | 0.46 | -0.46 |
| **EQ-5D-5L+R2** | 0.64 | 0.98 | 0.56 | -0.54 |
| ***VAS*** | 0.60 | 0.53 | 0.37 | -0.33 |
| ***WHODAS** | -0.60 | -0.84 | -0.40 | 0.34 |

*For WHODAS sum score was used ^i^ moving from level ***4 to 5*** there is no significant effect size
